# Medicine Pharmacy Interprofessional Exercise Pilot: Lessons Learned

**DOI:** 10.15694/mep.2018.0000066.1

**Published:** 2018-03-22

**Authors:** Samuel Miller, Elena A. Wood, Michael Fulford, Susan Fagan, Paul Wallach

**Affiliations:** 1 Medical College of Georgia; 2 University of Georgia

**Keywords:** Undergraduate Education, IPE, Medicine/Pharmacy

## Abstract

This article was migrated. The article was marked as recommended.

The Association of American Medical Colleges and the American Association of Colleges of Pharmacy with leaders of other health professions formed the Interprofessional Education Collaborative (IPEC) in 2009. Interprofessional education (IPE) is now a component of accreditation standards for academic programs in both medicine and pharmacy. While geographically separated, the Medical College of Georgia (Augusta University) and the College of Pharmacy (University of Georgia) have over 40 year history of collaboration but never intentionally added joint curricular offerings.To address IPE competencies, we developed and evaluated a medicine pharmacy collaborative exercise. Specifically, to compare the attitudes and perception toward interprofessional education and practice of students from two disciplines. An observational cross sectional study with 208 third year medical (M3) students and 108 third year pharmacy (P3) students was conducted in two consecutive academic years. Groups consisted of 6-8 M3 and 3-4 P3 students from all campuses across the state. The M3 student was to choose a patient they had seen who was taking at least 3 prescription medications. Objectives for students were to discuss the case (medications, side effects, cost, pharmacogenetics, drug-drug interactions, and cost effectiveness). A report of M3-P3 findings was to be submitted for grading by the Pharmacy faculty. Once the exercises were completed surveys were distributed and the de-identified data was analyzed in relation to IPEC competencies. The study was conducted in one academic year and 316 responses were obtained. Within medical students 48.2 % agreed and 21.2 % strongly agreed to consult with Pharm D in the future. Perception of leadership in the groups was shifted more toward pharmacy students (56.5% vs. 27.4%, p<0.0001). Students confidence in working with other professionals was improved more for pharmacy students than medical students (50.0% in P3 and 30.2% in M3 p =0.0014). Communication skills improved more in P3 than M3 (48.2% in P3 and 28.3% in M3, p=0.0043). More research is needed on equal adoption of IPE by medical and pharmacy students. Emphasis should be made on equal state of training (theory vs. clinical) on both sides with focus on working as a team and value of each team member. More direct involvement in patient care is needed with both M3 and P3 having face to face contact with patients and each other.

## Introduction

The World Health Organization (WHO) defines interprofessional education (IPE) as the following: “when students from two or more professions learn about, from, and with each other to enable effective collaboration and improve health outcomes.” [5]. Globally, for over three decades, health policy makers have identified the key role of IPE in improving health care systems and outcomes [ 6, 7]. Medicine and Pharmacy are uniquely suited to work together in IPE because of the close relationship we share in patient care. Health care reform promotes collaborative practice as one strategy for enhancing the quality and safety of health care [ 8, 9]. The interdependence between health profession education and collaborative practice is the theoretical basis for implementing IPE within all health profession curricula [ 10, 11].

The pharmacy program at The University of Georgia (UGA) and the medical program at The Medical College of Georgia (MCG), both parts of the State University System, and both with societal and accreditation mandates for interprofessional education sought to work together to identify and implement opportunities for interprofessional education between learners in each program and to seek other opportunities for collaboration, for example, in areas of research and service to the community. The Medical College of Georgia and the UGA College of Pharmacy have over 40 years history of collaboration but never intentionally added joint curricular offerings. The accrediting bodies (LCME, ACPE) have created new standards which require Medical and Pharmacy schools to incorporate Interprofessional Education as part of their curricular offerings [ 2, 3]. IPE requirements established by the different accreditation agencies are somewhat different. The programs that we implement together in some cases may meet a particular standard and in others may contribute toward further collegiality and promote other opportunities to collaborate. Medicine and Pharmacy are uniquely suited to work together in IPE because of the close relationship we have in patient care. M3 students have excellent patient assessment skills and P3 students have excellent knowledge of pharmacotherapy. Putting them together to discuss patients would allow both groups to learn from each other to improve patient care and understand different professional perspectives in patient care. In Georgia, the two programs have particular “common ground” as geography is shared across the state. Specifically, the Pharmacy program has the main campus in Athens at UGA and branch campuses in Augusta, Savannah, and Albany. The Medical program has a main campus in Augusta and regional campuses in Athens, Savannah, Albany, and Rome. In several cases, the program administrative offices are juxtaposed. For our IPE exercise we wanted to improve M3 and P3 students’ ability to obtain and organize patients and drug information, gathered from other health care professional students, in order to identify actual or potential drug related problems, to develop a collaborative plan to address each drug related problem and provide justification and document each problem.

The goals and objectives of the study were to state the existing Med-Pharm IPE exercises, evaluate student’s perception of IPE values and attitudes toward future inter-professional collaboration, and describe the Med-Pharm IPE leassons learned.

## Methods

This study was an observational cross-sectional study with the participants consisting of 208 third year medical students from Medical College of Georgia (M3) and 108 third year Pharmacy students from UGA College of Pharmacy (P3). All campuses, Augusta, Athens, Rome, Albany and Savannah, participated. The duration of the study was from January 2015 until April 30, 2015.

These students were divided into working groups of 6-8 students (Medicine and Pharmacy). One M3 student in each group selects a patient that they have had in their clerkship who is on three or more prescription drugs and succinctly presents the patient to the group. The presentation includes pertinent history, physical examination, physical test results, and diagnosis. The presentation and all other group interactions were either in person or by distance connection using Facetime, Google Hangout or Skype. The choice of communication was determined by each group dependent on the geographical location of the members of each group. All routine and prn medications were listed. The group discussed all therapies and identified potential drug related problems, assessed the appropriateness of the current medications on the basis of health conditions, indication, and the therapeutic goals of each medicine, and evaluated the effectiveness, safety and affordability of each medication. They discussed drug mechanisms of action, assessed medication-taking behaviors and adherence to each medication, and identified potential medication-related problems and evaluate the need for intervention. Finally, they evaluated these medications for interactions with each other or other common medications and are there any pharmacogenetic considerations. All recommendations were supported with published literature. Fifty eight group cases were submitted (2-page document by email) twice during the study time line. The P3 students were required to submit the completed patient case for 20% of their Pharmacotherapy grade in the Spring Semester. M3 students were not graded on their participation.

Course evaluation was performed electronically, using evaluation systems of two different universities and the results were combined. Surveys were developed for evaluation of the study for curriculum use. The questions on the survey were developed to evaluate the following: (A) These questions were addressed to both cohorts to evaluate their educational experience; (B) This group of questions were only submitted to medical students on their perception and attitudea; (C) This question was distributed in the survey to both cohorts to evaluate leadership skills.

Descriptive analysis was performed. All statistical analysis was performed using SAS 9.4 and statistical significance was assessed using an alpha level of 0.05. Chi-square tests were used to examine differences between medical and pharmacy students in the distribution of likert scale agreement responses for each questionnaire item. Only data that included in this analysis was where M3 and P3 responses presented.

The study received IRB approval from the IRB of Augusta University, project title 736319-1.

## Results and Discussion

### Results

#### A. Educational exercise outcomes

**Figure 1.**
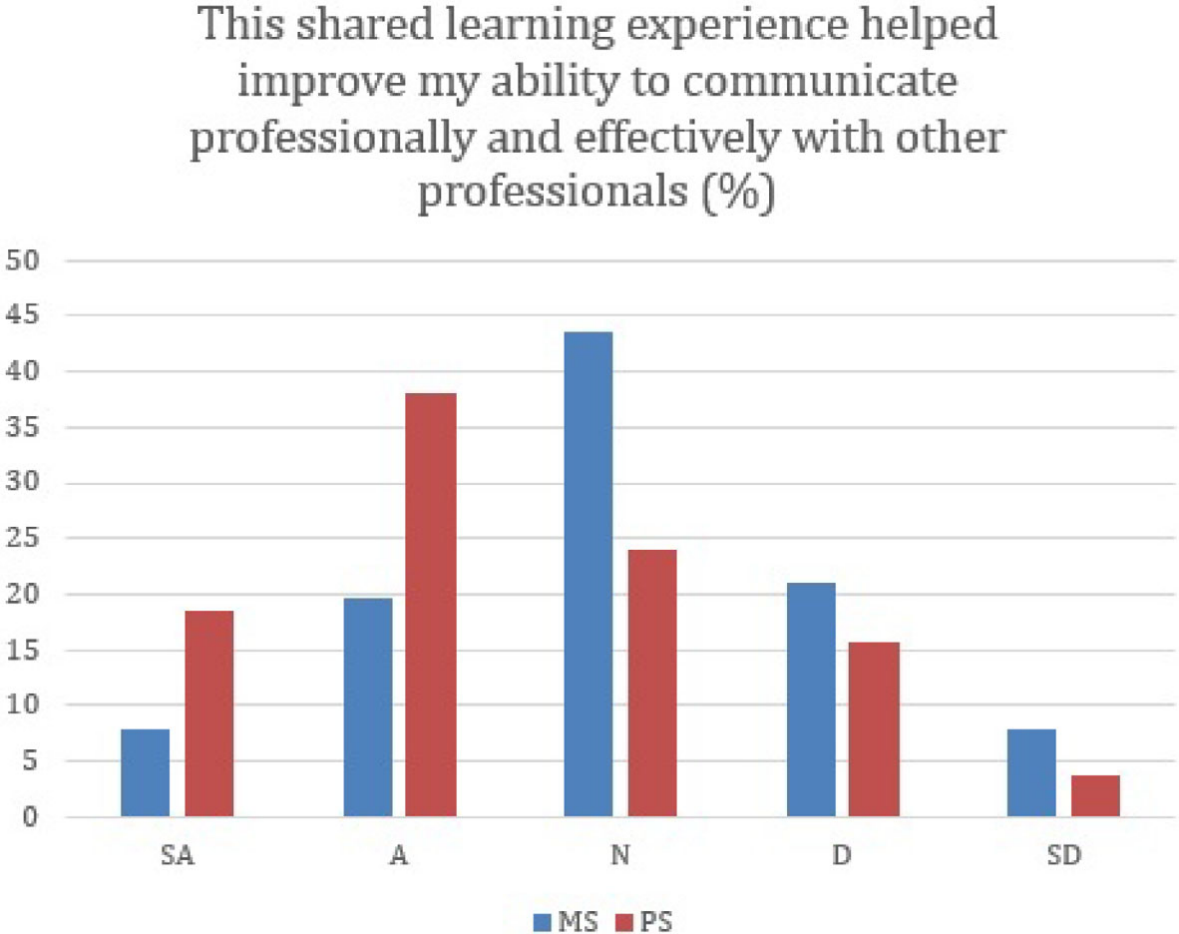
More Pharmacy Students (PS) agree and strongly agree than medical students (MS) that exercise added to understanding of IPE.

Five questions were asked regarding studets’ educational experiences ( Appendix 1). The groups felt that they did not function as a team in most cases and the P3’s felt as if they were the sole members of the team. The P3’s felt that the lack of a grade for the medical students resulted in less participation as a team member. Medical students were perceived as being too busy to participate in the team activities and the burden fell on either the pharmacy student or the other members of the group. Figure 1 presents the trend of medical students and pharmacy students responses distribution.

Pharmacy students have significantly higher percentages of strongly agree and agree than medical students on perception of (a) improved understanding of interprofessional practice as a benefit to patient care (P=0.002); (b) improved confidence in interprofessional setting (P=0.0014); (c) improved ability to communicate effectively and efficiaently with other prefessions (P=0.0043); (d) improved understanding of the role on the interprofessional team (P=0.0046).

**Figure 2.**
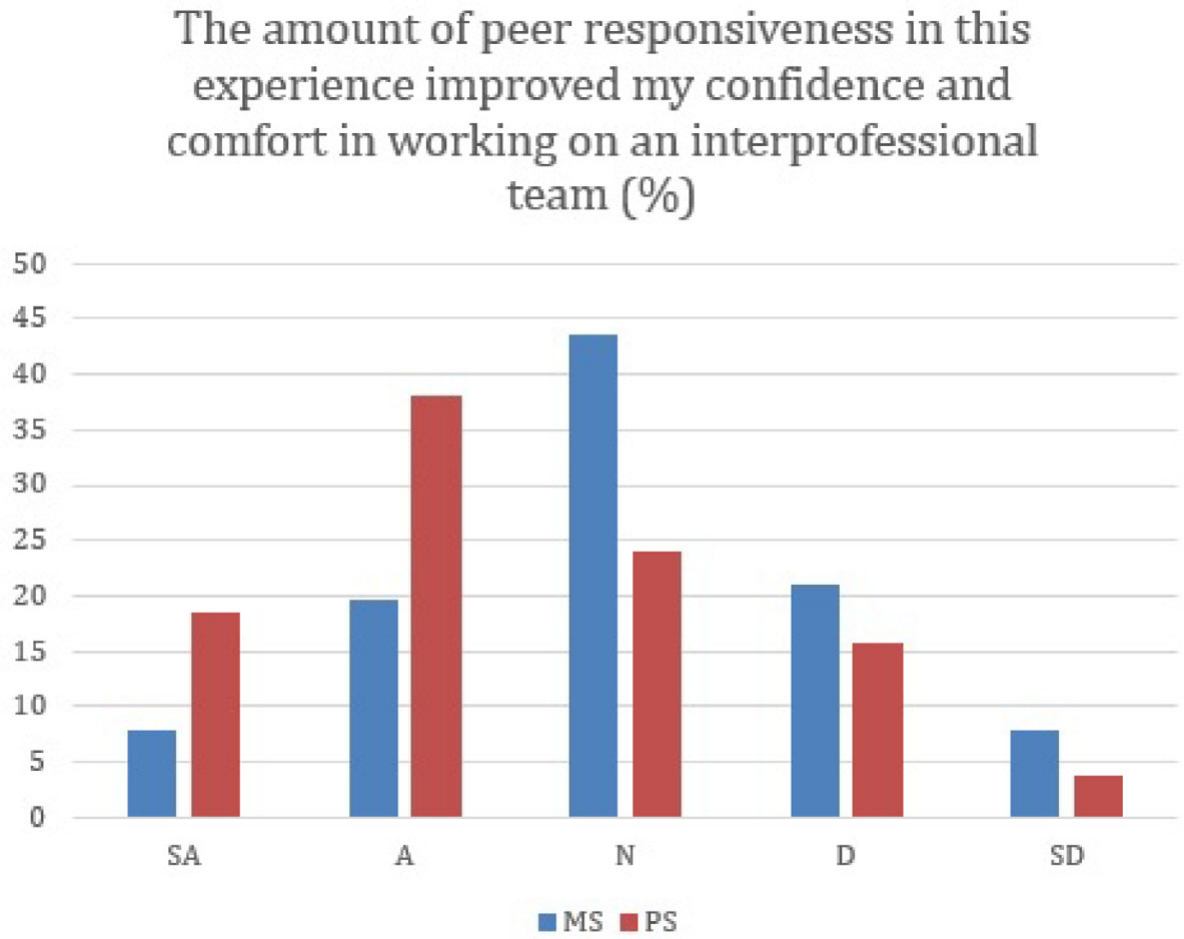
Improvement of Pharmacy students in working on an Interprofessional team

Medical student have significantly higher percentages of neutral to strongly disagree than pharmacy students in response to the peer responsiveless improved confidence and comphort working on an interprofessional team ( Figure 2) (P=0.041).

#### B. Medical Students’ perception

Four questions were asked medical students only ( Appendix 1). Out of 208 medical students 21% strongly agreed and 48 agreed to consult Pharm D again. On the ability to meet patient needs medical students majority either agreed (37%) or stay neutral (33%). Same number of students reached agreement or disagreement on more effective way to find drug related information: look by themselves or ask Pharm D (30% vs. 30%). More medical students reached agreement (36%) than disagreement (28%) on perception of working with Pharm D helped understanding of benefit to patient care.

#### C. Leadership perception

**Figure 3.**
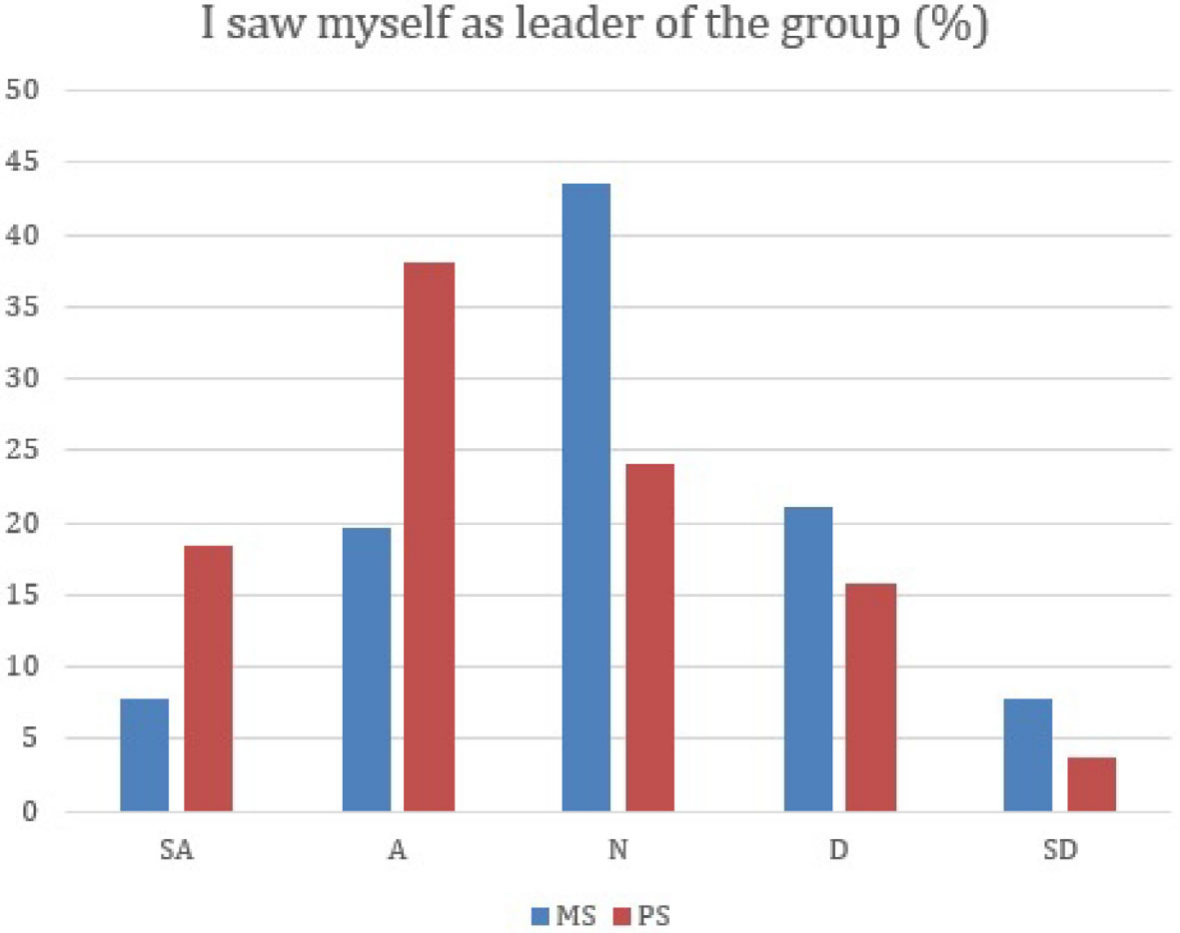
Medical and Pharmacy students’ responces on a leadership role.

Pharmacy students have significantly higher percentages of strongly agree and agree than medical students on a leadership role on the group ( Figure 3) (P<0.0001).

### Discussion

We believe IPE is important and have a novel state-wide construct for our MD program (AU) and Pharmacy program (UGA). We developed an exercise for all to work together. Concerned about a new program, we created something that we thought would introduce learners to each other digitally, not require in-person meetings, and would engage in the discussion of a patient.

The educational design of the exercise led to some outcomes that would lead us to redesign the educational excersise. For the pharmacy students, the exercise counted significantly toward their grade. For the medical students, they just needed to complete the exercise without being graded. Therefore, some medical students did not take it as seriously as it should have been. As a result, the pharmacy students saw themselves as leaders of the group more often than medical students. The percentage of neutral on average between the two groups was 20% higher in the group which had educational requirements demonstrates that external motivation exerts an influence on how serious the exercise was approached. This mean that without external motivation (grading) busy medical students do not actively involved into educational excersises.

The pilot study involved a patient that was only seen by the medical student and reported on after discharge. The pharmacy student never saw the patient, but relied on information provided by the medical student. Better outcomes might be expected when simultaneous exposure to the same patient with discussion of findings rather than patient case review.

The exercise involved contact between the two groups of students for only 1-2 hours on one or two occasions. Better outcome about perception of working with different disciplines might improve from working on different patients over a longer period of time. Non face to face encounters had a negative impact on students’ engagement in IPE.

The third year pharmacy students were still in the classroom whereas the third year medical students were clinical and the level of understanding of the exercise was different. Therefore, one of the ideas was to include fourth year pharmacy students and third year medical students as they both would be involved in clinical care of patients. Earlier intervention in the curriculum may improve the perception of the importance of team based care. This was a limited single exercise that was performed in an environment that did not include face-to-face interaction. It also centered on a patient who was only seen by a medical student, not the pharmacy students, and the patient had typically already been discharged from the hospital. Given the construct, not a surprise that opinions on team care were not as favorable as should be, yet, students agreed that they gained understanding, confidence, communication, roles, confidence, etc even with this very brief exercise that is not optimal in design. Further investigation is needed to understand Medical Students perception of the value of the exercise in team based care.

## Conclusion

An attempt was made to incorporate interprofessional education into the curriculum of two schools to evaluate the level of acceptance of working together for a common goal. More research is needed and a redesign of the study is needed for equal adoption of IPE by medical and pharmacy students. The experience that involved an one-time exercise for a few hours does not allow to improve significantly students’ perception of benefit of interprofessional practice for patient care. External motivation was a big factor in investment of time and energy into the exercise and should be taken into account then such educational interventions designed. Level of medical knowledge should be equivalent from all interprofessional groups to be meaningful excercise.

## Notes On Contributors

Samuel Jones Miller, PhD, MD provided educational intervention stategies, data collection and preparation and submission of paper.

Elena A. Wood, Phe, MD provided educational intervention, data collection and paper presention and editing.

Mike Fulford, PhD, provided data analysis and statistical analysis for Pharmacy. Assisted in data evaluation.

Susan Fagan, Pharm D, Study design, supervised study for School of Pharmacy, progress evaluation and data analysis.

Paul Wallach, MD, provided oversight of paper preparation, data analysis and educational evaluation.

## References

[1] Interprofessional Education Collaborative Connecting health professions for better care. (2017). https://www.ipecollaborative.org/about-ipec.html

[2] Accreditation Council for Pharmacy Education. Accreditation standards and guidelines for the professional program in pharmacy leading to the Doctor of Pharmacy Degree. (2011). https://www.acpe-accredit.org/pdf/Standards2016FINAL.pdf

[3] Functions and Structure of a Medical School. Standards for Accreditation of Medical Education Programs Leading to the MD Degree. (2016). http://lcme.org/publications/

[4] IPEC Core Competencies for Interprofessional Collaborative Practice. 2016 Update. https://www.tamhsc.edu/ipe/research/ipec-2016-core-competencies.pdf 10.1080/13561820.2019.1624513 31192744

[5] Centre for the Advancement of Interprofessional Education. Interprofessional Education: A Definition. 2002. updated 2011 Feb 10). https://www.caipe.org/

[6] World Health Organization . Continuing Education of Health Personnel. Copenhagen: WHO Regional Office for Europe(1976).

[7] World Health Organization . Framework for Action on Interprofessional Education and Collaborative Practice. 2009. (updated 2010 May 30)

[8] Schmitt M Blue A Aschenbrener CA Viggiano TR . Core competencies for interprofessional collaborative practice: reforming health care by transforming health professionals’ education. Acad Med. 2011;86(11);1351. 10.1097/ACM.0b013e3182308e39 22030650

[9] Reeves S Perrier L Goldman J Freeth D Zwarenstein M . Interprofessional education: effects on professional practice and healthcare outcomes (update). Cochrane Database Syst Rev. 2013;3;CD002213. 10.1002/14651858.CD002213.pub3 PMC6513239 23543515

[10] Interprofessional Education Collaborative Expert Panel. Core competencies for interprofessional collaborative practice: report of an expert panel. http://www.aacn.nche.edu/education-resources/percreport.pdf

[11] D’Amour D Oandasan I . Interprofessionality as the field of interprofessional practice and interprofessional education: an emerging concept. J Interprof Care. 2005;19(suppl 1):8–20. 10.1080/13561820500081604 16096142

