# Supplementary material for: Medicine Pharmacy Interprofessional Exercise Pilot: Lessons Learned
Source: MedEdPublish (2016). 2018 Mar 22;7:66. [Version 1] doi: 10.15694/mep.2018.0000066.1 (PMC10712014; doi:10.15694/mep.2018.0000066.1)
Supplement: Peer review history

# Reviewer report: Reviewer response for version 1

**DOI:** 10.21956/mep.19531.r28911

**Published:** 2019-07-22

**Authors:** Trevor Gibbs

**Affiliations:** 1 AMEE

This review has been migrated. The reviewer awarded 4 stars out of 5 A well written paper covering the topic of IPL and well structured. The paper pointed out the importance of having pre-determined learning objectives for both groups. I liked the way that the authors described both the positive and negative outcomes of the paper and the way to move forward with their initial findings, which I hope they pursue. .

Reviewer Expertise:

NA

No decision status is available

# Reviewer report: Reviewer response for version 1

**DOI:** 10.21956/mep.19531.r28913

**Published:** 2019-03-04

**Authors:** Ken Masters

**Affiliations:** 1 Sultan Qaboos University

This review has been migrated. The reviewer awarded 3 stars out of 5 An interesting read and a useful pilot for IPE, which will always present challenges. The grading / not grading is a typical example of problems caused by different requirements of courses that are brought together. The different levels of expertise are also typical of IPE problems.Some issues:• With an opening exploratory study such as though, however, it would have been more useful if the researchers had allowed for qualitative research also. At the moment, they have answers, but only to the questions that they anticipated and asked. There may be many more issues that they have not foreseen, and which could be discovered only with qualitative research. Interviews or focus group would have been advised; at the very least, a general box asking for other comments in the survey form would have helped.• I do not see any indication of demographics. As demographics may impact of the student experience, it would have been worthwhile if the authors had gathered this information, and then analysed in light of those data.Small issues: • “close relationship we share in patient care” and “because of the close relationship we have in patient care.” It is not clear here who the “we” is. This needs clarification or correction.• A few small typographical errors, e.g. “Medical student have”; these should have been cleaned up with good proofing.

Reviewer Expertise:

NA

No decision status is available

# Reviewer report: Reviewer response for version 1

**DOI:** 10.21956/mep.19531.r28912

**Published:** 2018-03-22

**Authors:** Mary Louise Wright

**Affiliations:** 1 University of Wollongong

This review has been migrated. The reviewer awarded 4 stars out of 5 Aims and objectives - clear and highly relevant to IPEStudy design - neat and well designed. It would have been nice to know how often groups met (although I suspect this varied) and whether medical students, although not graded, had to participate as an expected activity of their curriculum. Results: clearly explained and well represented. Were the validity and consistency of the survey looked at? The questionnaire is included in this presentation of the study and is short, easy to use and looks to be well designed for participant ease of use. The results are interesting and highly relevant in the field of IPE. Medical student involvement and buy in is always tricky - mainly due to timetabling or packed curriculum - but this study describes and evaluates a clever model and although Pharmacy students demonstrated greater commitment and educational value form the exercise there was nevertheless medical student agreement that they would use Pharm D consults in future. Conclusions/ discussion: Some pertinent observations made. Thank you - as a person involved in IPE projects with senior medical and allied health students I both found this paper relevant and enjoyed reading it.

Reviewer Expertise:

NA

No decision status is available
